# Position‐specific induction of ectopic limbs in non‐regenerating blastemas on axolotl forelimbs

**DOI:** 10.1002/reg2.10

**Published:** 2014-02-16

**Authors:** Catherine McCusker, Jeffrey Lehrberg, David Gardiner

**Affiliations:** ^1^ Department of Developmental and Cell BiologyUniversity of CaliforniaIrvineUSA

**Keywords:** Axolotl, limb regeneration, positional information, reprogramming, retinoic acid

## Abstract

Ectopic retinoic acid (RA) has been hypothesized to reprogram the positional identity of cells in developing and regenerating limbs to a single positional value corresponding to the posterior‐ventral‐proximal (PVPr) position on the limb. We tested this hypothesis by using RA to reprogram the information of blastema cells that were induced to form at different positions around the limb circumference. We observed that RA treatment of blastemas in anterior and dorsal locations, but not posterior and ventral locations, resulted in the induction of complete ectopic limbs. These position‐specific differences in limb induction are probably due to differences in the positional disparity between the RA‐reprogrammed blastema cells and the cells at the periphery of the wound. These observations are consistent with the hypothesis that RA treatment reprograms the information in blastema cells to the PVPr position on the limb, since anterior and dorsal positions have the largest disparity and posterior and ventral have the smallest disparity from the PVPr identity.

## Introduction

The goal of regenerative biology is to understand the mechanisms driving the regeneration of complicated biological structures so that they can be recapitulated to stimulate a regenerative response in humans. Urodele amphibians serve as an excellent model to study the mechanism of regeneration in a vertebrate system because they have the amazing capacity to regenerate complicated body structures including their jaws, limbs, and tails (Ferretti 1996; Tanaka 2003; Han et al. 2005). Our laboratory has used a gain‐of‐function assay (the accessory limb model, ALM) in the axolotl (*Ambystoma mexicanum)* to study the role of different cell types and signaling pathways during limb regeneration. The ALM is based on the observation that ectopic limbs can grow from a wound on any region of the limb provided a severed nerve is present and that cells from a different position on the limb axis are grafted into the wound site (Endo et al. 2004). Thus, the ALM can be used to test molecules that are either involved in (1) the neurotrophic response (Singer 1974) or (2) establishing positional diversity in the limb regenerate (French 1978; Bryant et al. 1981).

The objective of the current study was to use the ALM to study whether the reprogramming of positional information of blastema cells could elicit a regenerative response in blastemas that normally are non‐regenerative. Non‐regenerative blastemas, which undergo the initial stages of blastema formation but eventually stop growing and are re‐integrated into the limb without generating new structures, are induced to form by deviating a nerve to a wound on the side of the limb. Our hypothesis is that non‐regenerative blastemas do not generate limb structures because they lack the diversity of positional information required to generate a complete limb field (Endo et al. 2004). To test this hypothesis we utilized the reprogramming capacity of exogenous retinoic acid (RA), which has been well documented in its ability to reprogram the positional information in developing and regenerating limbs but not in differentiated cells (Maden 1983; Stocum and Thoms 1984; Eichele et al. 1985; Kim and Stocum 1986; Larsen and Janners 1987; Ludolph et al. 1990; Noji et al. 1991; Tickle 1991; Wanek et al. 1991; Bryant and Gardiner 1992).

We observed that only anterior and dorsally located blastemas formed complete ectopic limbs when treated with RA. In contrast, posterior and ventrally located blastemas had a minimal capacity to form ectopic structures when exposed to exogenous RA. These observations suggest that exogenous RA can substitute for a tissue graft in the ALM because it results in the formation of positional diversity between the cells in the wound site and the RA‐reprogrammed blastema. Furthermore, these results demonstrate for the first time the chemically induced formation of ectopic limbs in the absence of a tissue graft and provide a useful tool for the future mapping of positional identities within the limb.

## Results

### Retinoic acid treatment induces the formation of ectopic limbs from “non‐regenerating” anterior blastemas

**Figure 1:**
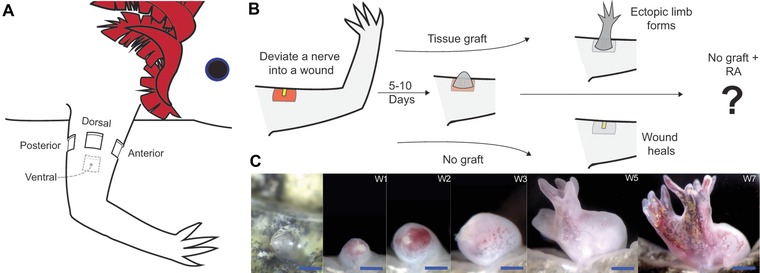
Treatment of a “non‐regenerative” anterior wound with retinoic acid (RA) induces the formation of ectopic limbs. (A) Diagram of an axolotl forelimb indicating the positions at which wounds were made around the limb circumference. (B) Diagram illustrating the experimental procedures performed on anterior wound sites. An anterior wound site with a deviated nerve forms an ectopic blastema within 5−10 days. Without a posterior skin graft, the ectopic blastema integrates into the limb and the wound heals over without forming an ectopic limb (Endo et al. 2004). If a skin graft from the posterior side of the limb is grafted into the wound site, an ectopic limb is generated (Endo et al. 2004). Ectopic blastemas without a graft (non‐regenerating) were treated with RA to determine whether RA can replicate the positional confrontation normally induced by the posterior tissue graft. (C) Images of the same ectopic limb induced by RA treatment of an anterior ectopic blastema taken over a period of 7 weeks. The first image is the blastema on the day of RA treatment. The blastema forms an amorphous mass that eventually forms two well patterned ectopic limbs. These images are representative of what was observed in 10 of 20 ectopic structures that eventually formed paired ectopic limbs from an RA‐treated anterior wound (see Table 1). Blue scale bars are 1 mm in length.

Previous studies have shown that ectopic blastemas are induced when a nerve is surgically deviated to an anterior wound site (Maden and Holder 1984; Endo et al. 2004). These blastemas eventually will cease to grow and then integrate into the existing limb tissue without generating ectopic limb structures unless tissue with opposing positional information (posterior) is grafted into the wound site. If tissue from the posterior side of the limb is grafted into the anteriorly located wound, the positional disparity between cells of the graft and the wound site stimulates the intercalation of a complete limb field, and an ectopic limb is generated (Fig. 1A, B) (Endo et al. 2004).

Ectopic RA has been hypothesized to reprogram the positional identity of cells in developing and regenerating limbs (Noji et al. 1991; Wanek et al. 1991) to a single positional value corresponding to the posterior‐ventral‐proximal (PVPr) position on the limb (Bryant and Gardiner 1992). We tested this hypothesis by using RA to reprogram the identity of cells in non‐regenerating anteriorly located blastemas to generate sufficient positional disparity (i.e., cells with posterior positional identity) to induce ectopic limb structures (Fig. 1). Within 2 weeks following RA exposure, most of the anterior blastemas had increased in size (Fig. 1C). Many of the blastemas formed a bulbous mass, as was previously observed in RA‐treated frog tail blastemas (Mohanty‐Hejmadi et al. 1992), which eventually formed limb structures. Most (71%) of the RA‐treated anterior blastemas generated skeletal elements (Table 1). Of the blastemas that generated ectopic structures, 50% of them formed paired limbs.

**Table 1:** Limb phenotypes resulting from RA treatment of animals with ectopic blastemas

Wound location	Total surgeries performed	Surgeries that developed blastemas*	Blastemas that formed ectopic structures	Single cartilage element**	Multiple symmetrical cartilage elements**	Single limb**	Paired limbs**
Anterior	30	28 (93%)	20 (71%)	4 (20%)	6 (30%)	0	10 (50%)
Dorsal	34	18 (53%)	9 (50%)	2 (22%)	1 (11%)	2 (22%)	4 (44%)
Posterior	26	26 (100%)	5 (19%)	4 (80%)	1 (2%)	0	0
Ventral	21	18 (86%)	1 (6%)	1 (100%)	0	0	0

*Percentage of surgeries that developed blastemas.

**Percentage of ectopic blastemas that formed structures.

**Figure 2:**
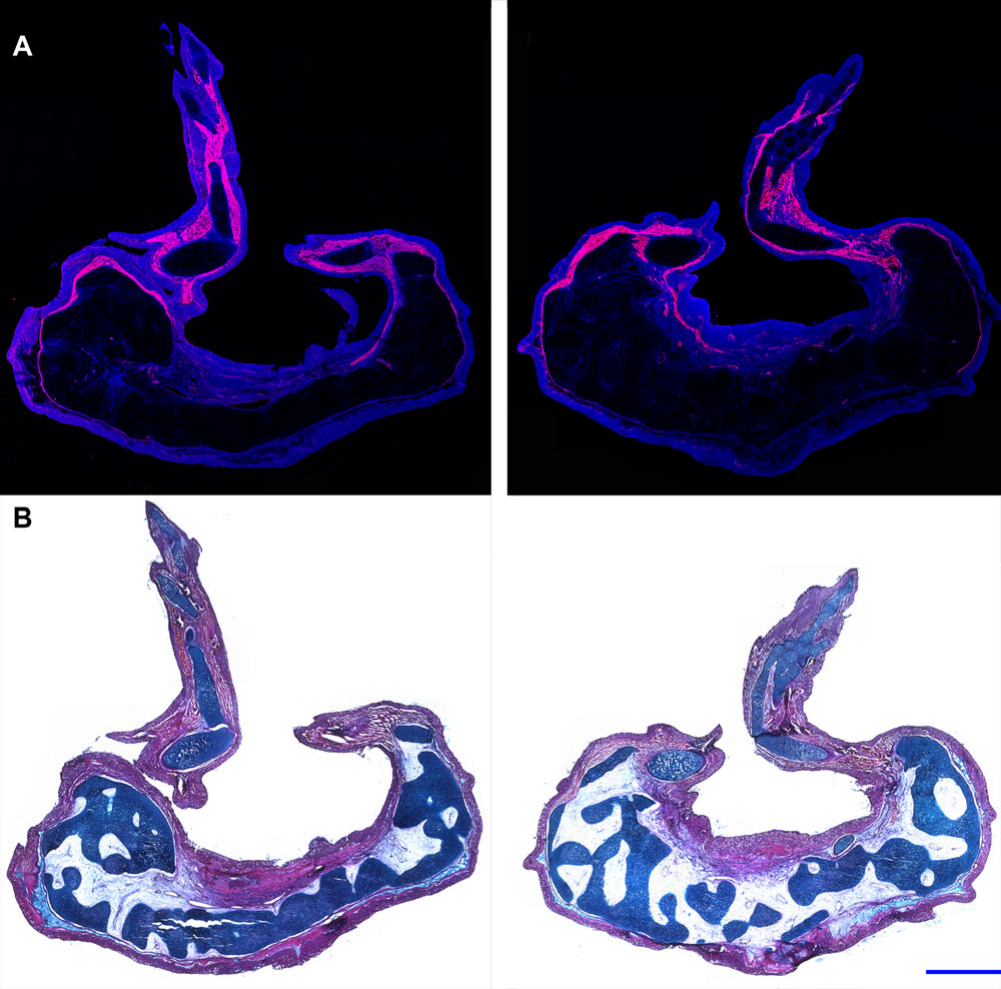
Histology of RA‐induced paired limbs from an anterior wound site. Histological analyses were performed on sections that transected each of the two ectopic limbs that grew from an RA‐treated anterior wound site, harvested 10 weeks post‐treatment. Two complete limbs, including the zeugopod, stylopod, and autopod, formed from this experimental manipulation. A large mass of tissue formed proximal to the limb structures. (A) Fluorescent images were obtained of the cryosectioned limbs stained with DAPI (blue) and phalloidin‐rhodamine (red). Muscle tissue, rich in F‐actin (stained with phalloidin‐rhodamine), was distributed throughout the more distal limb structures but was not observed in the mass of tissue located proximally. (B) Sections were stained with eosin Y, hematoxylin, and alcian blue for histological analysis. The proximal mass of tissue predominantly differentiated into connective tissue and cartilaginous elements (dark blue) that could not be identified as corresponding to skeletal elements that are part of the normal limb pattern. Blue scale bars are 1 mm in length.

**Figure 3:**
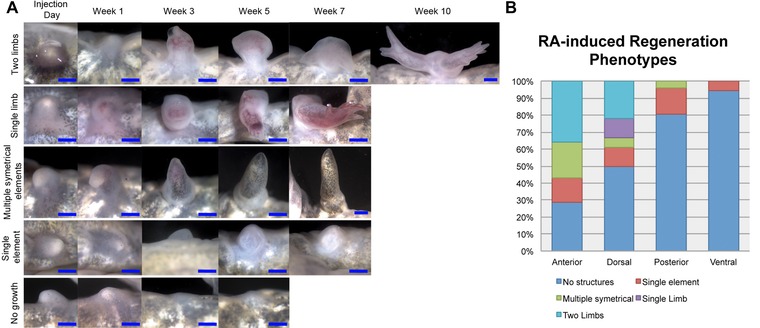
RA‐induced regeneration phenotypes are position‐specific. Quantification of the regeneration phenotypes for RA‐induced blastemas. Upon the completion of differentiation, the ectopic outgrowths were collected and stained for bone and cartilage in whole mount preparations in order to analyze the presence of skeletal elements in the regenerate. From our observations of these skeletal preparations, we divided the regeneration phenotypes into five categories: (1) no ectopic structures; (2) a single skeletal element; (3) multiple (jointed) symmetrical skeletal elements; (4) a single limb; or (5) two paired limbs (also see Table 1). (A) Examples of blastemas that exhibited the five different regeneration phenotypes that were quantified in this study. Images were taken of the blastemas starting the day of RA injection and ending when the tissue in the wound site had completely differentiated (determined by the formation of mature skin). (B) The histogram represents the percentage of ectopic blastemas located on the anterior, dorsal, posterior, or ventral axis that differentiated into each phenotype. Only the wounds located on the anterior or dorsal axis generated one or two paired limbs when treated with RA (also see Table 1). Blue scale bars are 1 mm in length.

There was some variability on where the double limbs formed from the bulbous mass. Some of the paired limbs formed from blastema‐like bumps on opposite ends of the mass, while others formed from blastema‐like bumps that arose from the same region of the mass (compare Fig. 1 with Figs 2 and 3A). In a few samples, we also noticed that the double blastemas developed at slightly different rates (Fig. 1C, week 5 image), suggesting that the paired limbs developed independently of each other. The limb morphology of the paired limbs was usually normal, although we did notice that paired limbs that developed from blastemas that formed close together often had patterning defects, such as skeletal fusions and missing digits.

In the RA‐treated blastemas that formed limbs, we observed that the skeletal elements in the basal region of the ectopic growth were not integrated with the humerus at the site of the wound. This lack of integration of the skeleton of ectopic limbs was also observed with ectopic limbs induced by a posterior skin graft (Endo et al. 2004; Satoh et al. 2007). In many cases the entire ectopic growth (both skeletal elements and associated soft tissues) eventually became connected to the host arm site only by a thin bridge of soft tissues. This phenotype is comparable to what has been reported in regenerating frog tail blastemas that were induced to form ectopic limbs when exposed to exogenous RA (Mohanty‐Hejmadi and Crawford 2003).

In addition we observed that the skin of the bulbous proximal region of RA‐treated ectopic blastemas had few or no pigment cells, whereas the ectopic limb structures that formed more apically were pigmented (Fig. 1C). The presence of pigment cells is considered to be the final step in skin maturation (Seifert et al. 2012) suggesting that the bulbous mass might be composed of undifferentiated cells; however, that was not the case (Fig. 2). The more apical limb structures exhibited skeletal patterns that were typical of normal limbs and contained muscle and cartilage. In contrast, the bulbous proximal region lacked muscle, but contained other differentiated limb tissues including cartilage and connective tissue. Although well differentiated, the basal cartilaginous elements could not be identified as corresponding to skeletal elements that are part of the normal limb pattern (Fig. 2). Despite the absence of pigment cells, the skin of the basal bulbous masses appeared to be well differentiated as evidenced by the presence of the basement membrane and gland cells (Fig. 2).

As with normal limbs, the pigment in the skin of the apical ectopic limbs was asymmetrically distributed (e.g., Fig. 1C) such that the pigment was restricted to approximately half of each limb. All paired limbs exhibited a pigment pattern in which the surfaces of the limbs apposed to each other were pigmented and the opposite surfaces were not pigmented. In normal limbs, pigmentation is restricted to the dorsal half of the limb, and thus ectopic paired limbs appear to exhibit a double‐ventral phenotype with their dorsal surfaces facing each other and their ventral surfaces facing outwards (Fig. 1C).

### Retinoic acid induction of ectopic limbs occurs in anterior and dorsal but not posterior and ventral located ectopic blastemas

As noted above, exogenous RA is hypothesized to reprogram the positional information of cells in the blastema or the limb bud to a ventral as well as a posterior identity (Bryant and Gardiner 1992). Since anterior and dorsal locations would have the largest positional disparity relative to the PVPr positional information induced by RA, a prediction of this hypothesis is that RA treatment will induce the formation of ectopic limb structures at high frequency from dorsal blastemas (the position opposite from ventral), but at much lower frequencies from posterior or ventral ectopic blastemas (with the same positional information as the RA‐reprogrammed blastema cells). By similar reasoning, blastemas at anterior/dorsal positions should form more complex, ectopic limb patterns compared with RA‐treated blastemas at posterior/ventral positions.

**Figure 4:**
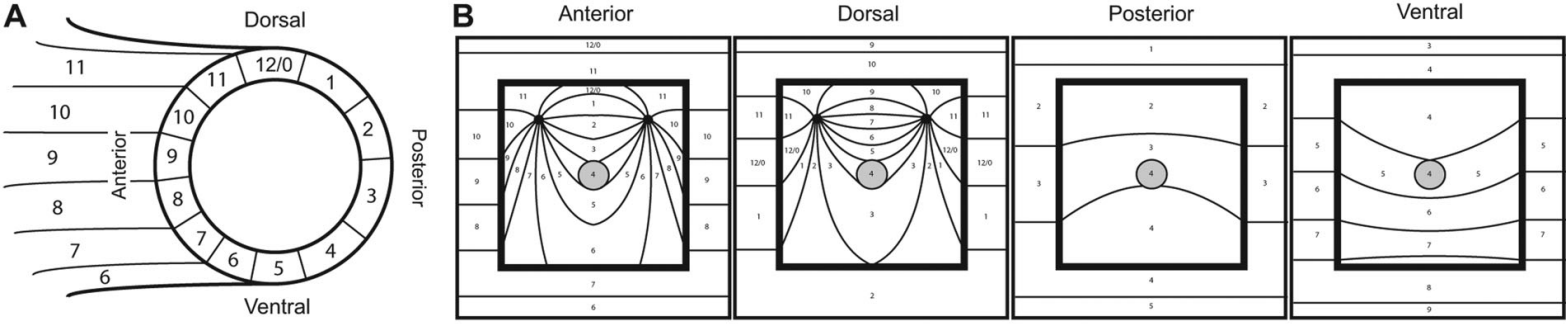
Model of RA‐induced supernumerary limbs from ectopic blastemas. (A) Diagram of an amputated limb showing the distribution of positional information around the limb circumference as described in Bryant et al. (1981) and Bryant and Iten (1976). The experiment was performed on wound sites that were made on the most anterior (“9”), dorsal (“12/0”), posterior (“2/3”), or ventral (“5”) locations. (B) Models representing the positional interactions between the RA‐reprogrammed blastema (gray circle with the “4” coordinate) and the cells in the surrounding wound margin. The boundary of the square wound is indicated by heavy‐weighted lines. The thin lines and corresponding numbers on the outside of the square correspond to the distribution of circumferential positional information in the host limb as illustrated in (A). The thin lines and corresponding numbers inside the square wound are the predicated positional values that would be intercalated as a result of the interactions between the reprogrammed blastema cells (“4”) and the surrounding host cells. When the RA‐treated blastema is located in anterior and dorsal wound sites, the positional disparity is sufficient to induce the formation of two complete limb axes. In contrast, the interaction between the reprogrammed blastema cells with posterior or ventral host cells does not generate enough positional diversity to induce ectopic limb axes.

As predicted (Table 1, Figs 3, 4), dorsal RA‐treated blastemas also formed ectopic structures at a high frequency (anterior, 71%; dorsal, 50%). In addition to relatively simple cartilaginous structures, most of these blastemas formed either one (dorsal, 22%) or two, paired ectopic limbs (anterior, 50%; dorsal, 44%). In contrast, very few of posterior or ventrally located blastemas formed ectopic structures (posterior, 19%; ventral, 6%), and except for one blastema with multiple symmetrical elements (posterior, 2%) these ectopic structures were limited to a single cartilage element.

## Discussion

### Model of positional interactions induced by retinoic acid

Whereas the region‐specific induction of ectopic limbs is predicted by the hypothesis that RA converts cells to a PVPr identity (Bryant and Gardiner 1992), the complexity of the induced pattern of the ectopic limbs is predicted by the polar coordinate model (PCM) (French et al. 1976; Bryant et al. 1981). The PCM hypothesizes that, when cells with disparate positional information interact, growth is stimulated and the new cells adopt a positional identity that is intermediate between the original cells, a process referred to as “intercalation” (French et al. 1976). Thus new structure and pattern are formed (intercalated) until the normal pattern is restored. By this view, when all the information of the circumferential limb axes is present (“complete circle” = anterior + posterior + dorsal + ventral) an entire new limb will be formed (Fig. 4A) (French 1978; Bryant et al. 1981).

We modeled the predicted intercalary interactions between RA‐reprogrammed blastema cells and host cells surrounding the wound margin to visualize interactions that would or would not induce a complete circle of positional information (Fig. 4B). We first represented the limb circumference as a clock face with 12 positions (details in Fig. 4A) (French et al. 1976; Bryant et al. 1981). By this view, RA would reprogram blastema cells to position “4” (posterior/ventral). For blastemas in posterior/ventral positions, RA reprogramming of cells is predicted to result in little or no alteration in the normal pattern of distribution of positional information (Fig. 4B). In contrast, the intercalation of cells with positional information that is intermediate between value “4” (newly reprogrammed cells) and the surrounding host cells leads to the formation of two complete circles of positional information in both anterior and dorsal RA‐treated blastemas (Fig. 4B). We presume that predicted complete circles of positional information correspond to the observed supernumerary limbs that were induced by RA treatment of anterior and dorsal blastemas (Table 1, Fig. 3). In addition to predicting whether or not supernumerary limbs would be induced, the PCM also predicts that the supernumerary limbs will have mirror symmetrical patterns. Although the steps in formation of the final supernumerary limbs presumably are complex, the final pattern of the double limbs that formed from anterior wounds all appeared to have double‐ventral handedness with the unpigmented ventral sides facing away from each other (Fig. 4B).

### Potential mechanism of RA‐induced positional reprogramming in blastema cells

The effect that ectopic RA has on the positional program in blastema cells presumably is dependent on expression of the correct retinoic acid receptor (RAR) in cells that are capable of having their positional information reprogrammed. A number of RAR isoforms have been detected in the limb blastema and one of these isoforms, RAR**‐**δ_2,_ appears to be responsible for positional reprogramming in the limb blastema (Ragsdale et al. 1989, 1992, 1993; Pecorino et al. 1996). However, exposing cells that express RAR**‐**δ_2_ to ectopic RA is not enough for positional respecification. Ectopic RA only appears to affect the positional information in undifferentiated blastema cells (Niazi et al. 1985), yet similar amounts of RAR**‐**δ_2_ are expressed in both the mature limb and the blastema (Ragsdale et al. 1993).

Our hypothesis is that ectopic RA reprograms the positional information in blastema cells because they are positionally plastic (i.e., are undifferentiated). Recent studies have shown that cells of the early blastema and the apical tip of the late blastema, both of which can be reprogrammed by RA, are positionally plastic and adopt the region‐specific molecular fingerprint of a new host environment (McCusker and Gardiner 2013). In contrast, mature stump tissue and the basal region of the late blastema (which is differentiating) are not positionally plastic (McCusker and Gardiner 2013) and are refractory to the effects of RA. Since positional information is epigenetically encoded in adult cells (Rinn et al. 2006), and the expression of epigenetic modifiers is essential for regeneration (Stewart et al. 2009), it is possible that the epigenetic modifications render region‐specific genes susceptible to positional reprogramming. RA signaling is upstream of a number of region‐specific molecules, including multiple *Hox* genes, *Tbx* genes, *Prod1*, and *Meis1/2* (Simon and Tabin 1993; Mullen et al. 1996; Mercader et al. 2005; Wang et al. 2006; Kumar et al. 2007). Thus, it is possible that the “open” epigenetic state of some of these genes renders their promoters accessible to activated RAR**‐**δ_2_ in blastema cells that have been treated with RA. However, there is much to learn about the epigenetic state of positional information and how new positional information is programmed in blastema cells. Testing which epigenetic states are sensitive to positional reprogramming by exogenous RA may bring some answers about the nature of positional plasticity.

### Chemical induction of regeneration

Regeneration is a stepwise process that can be induced experimentally by signals from a deviated nerve and from grafted skin cells with differing positional information (Endo et al. 2004). Recently, a cocktail of growth factors (GDF‐5, FGF2, and FGF8) has been identified that can substitute at least in part for signals from a deviated nerve leading to blastema formation (Makanae et al. 2013). In the present study we have demonstrated that RA treatment can substitute for grafting of posterior skin to an anterior wound (presumably as well as grafting of ventral skin to a dorsal wound) in order to provide the subsequent signals required for ectopic limb regeneration. Studies focused on optimizing the combinatorial delivery of growth factors and RA treatment in order to induce regeneration of an ectopic limb through specific signaling molecules are in progress.

## Materials and Methods

### Animal husbandry

This study was carried out in accordance with the recommendations in the Guide for the Care and Use of Laboratory Animals of the National Institutes of Health. The experimental work was approved by the Institutional Animal Care and Use Committee of the University of California Irvine.

All of the experiments in this study were performed on wild type Mexican axolotls (*Ambystoma mexicanum*) measuring approximately 5−8 cm snout to tail tip (3−4 cm snout to vent). Experimental animals were either spawned at UC Irvine or obtained from the Ambystoma Genetic Stock Center at the University of Kentucky. Animals were anesthetized using a 0.1% MS222 solution (ethyl 3‐aminobenzoate methanesulfonate salt, Sigma, St. Louis, MO, USA), pH 7.0.

### Nerve deviation surgery

The induction of ectopic blastemas was performed as described previously (Endo et al. 2004). In brief, the brachial nerve bundle was deviated into a square wound site (1−2 mm on a side) on the most anterior, dorsal, posterior, or ventral region of the proximal forelimb (stylopod). Given the ventral location of the brachial nerve, it was technically more challenging to deviate the end of the severed nerve to wounds created on the opposite side of the limb (dorsal), resulting in a reduced frequency of ectopic blastema induction (Table 1). Mid‐bud stage blastemas typically formed 7−10 days after surgery.

### Retinoic acid treatment

Ectopic blastemas were allowed to develop until mid‐blastema stage (approximately 7−10 days), at which point animals were injected intraperitoneally in the flank with RA (150 μg/g of body weight as described in Niazi et al. 1985). Animals were kept in the dark for 2 days following the injection to minimize the photo‐inactivation of RA. Live images of blastemas were taken on a weekly basis starting on the day of RA injection and continuing until fully regenerated skin had formed over the regenerate or wound site, at which time the limbs were collected for further analysis.

### Histology staining

Tissues were fixed in 3.7% paraformaldehyde. Tissues for histological analysis were prepared for cryosectioning. For fluorescent histology, sections were stained with phalloidin‐rhodamine for F‐actin and 4’,6‐diamidino‐2‐phenylindole (DAPI) for nuclei. The sections were stabilized with Vectashield mounting medium (Vector Laboratories, Burlingham, CA). Fluorescent images were obtained using a 20× objective on a Zeiss LSM780 (two‐photon) confocal microscope. Tissue sections were also stained with 0.03% Alcian blue/0.1% HCl/70% ethanol for 30 min, followed by standard hematoxylin and eosin Y staining.

### Whole mount bone and cartilage staining and phenotype scoring

For the phenotype analysis presented in Table 1 and Figure 3(B), the presence and complexity of ectopic skeletal elements were assessed by the use of whole mount bone and cartilage staining as described in Horton and Maden (1995). Representative images of the skeletal staining on samples that exhibit each of the different phenotypes from blastemas treated with RA are shown in Figure S1.

Samples that formed two blastema‐like structures that either developed into independent limbs or had skeletal fusions in the stylopod but formed independently from the elbow‐joint distally were scored as “paired limbs.” The “single limb” phenotype was scored on samples that resulted in the formation of a single limb with stylopod, zeugopod, and autopod elements. One of the single limbs had extra digits, and the other was missing digits. Samples that were scored as “multiple symmetrical elements” had multiple skeletal elements that were separated by joints. The “single cartilage element” samples had one, small, spherical or ovoid shaped cartilage element that had no joints and did not display any obvious characteristics of a particular limb skeletal element. Blastemas that resulted in “no growth” after treatments with RA did not form a bulbous mass and eventually integrated into the limb.

## Supporting information

Disclaimer: Supplementary materials have been peer‐reviewed but not copyedited.

**Figure S1**. Representative images of whole mount skeletal preparations on ectopic skeletal elements from RA‐treated blastemas.
