# Supplementary figures and images for: Position‐specific induction of ectopic limbs in non‐regenerating blastemas on axolotl forelimbs
Source: Regeneration (Oxf). 2014 Feb 16;1(1):27–34. doi: 10.1002/reg2.10 (PMC4906668; doi:10.1002/reg2.10)

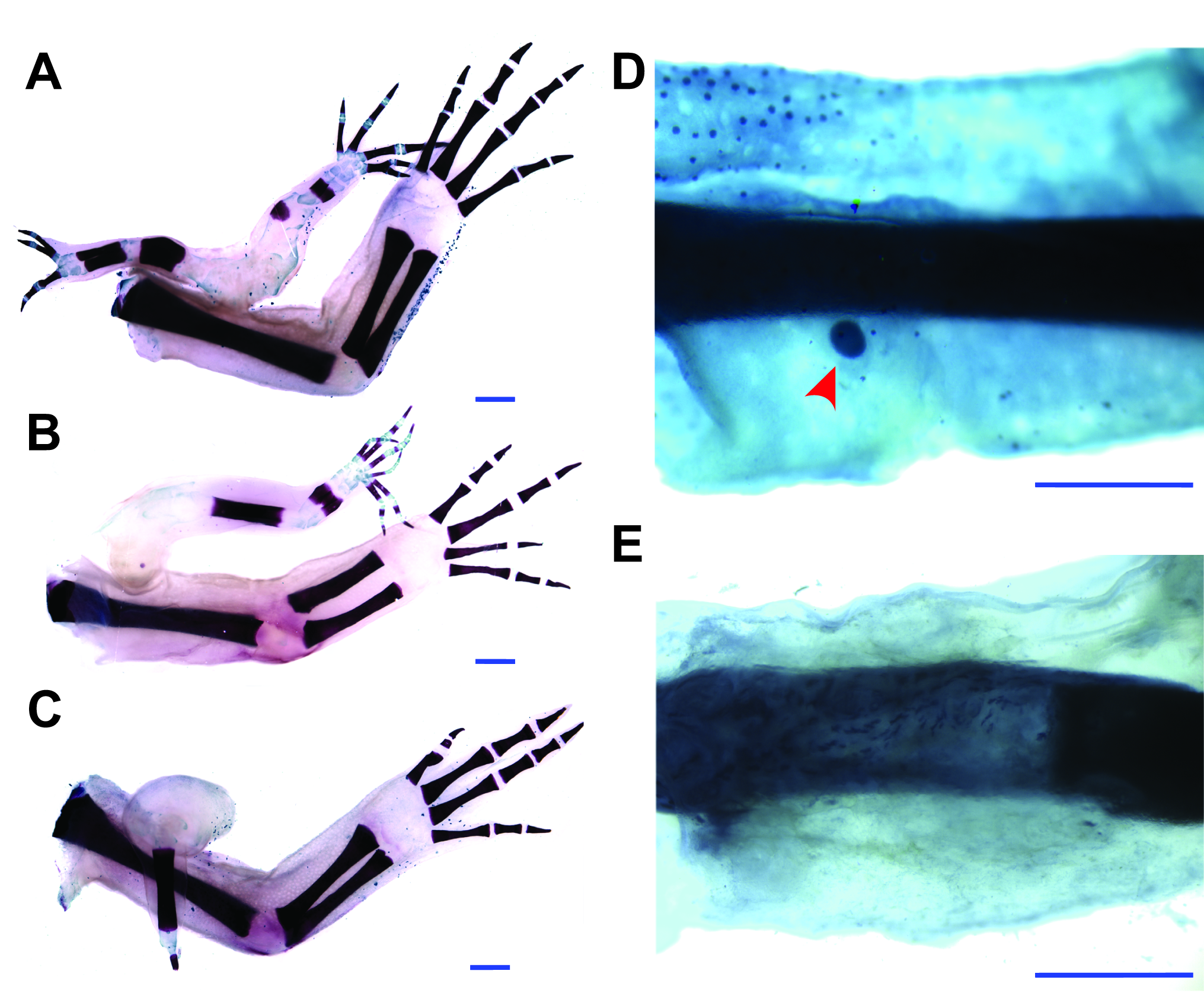

Supplement: Supplementary file 1 — Figure S1. Representative images of whole mount skeletal preparations on ectopic skeletal elements from RA‐treated blastemas. [file REG2-1-27-s001.tif]
